# Complete chloroplast genome analysis of *Hypopterygium flavolimbatum* Müll. Hal. (Hypopterygiaceae, Bryophyta)

**DOI:** 10.1080/23802359.2025.2535629

**Published:** 2025-07-22

**Authors:** Zheng-Kai Ye, Guo-Yong Yang, Ye Tang, Qin-Yu Liu, Li-Hua Chen, Xiang-Hua Fang

**Affiliations:** a Jingning County Ecological Forestry Development Center, Lishui, China; b College of Forestry Science and Technology, Lishui Vocational & Technical College, Lishui, China

**Keywords:** Bryophyte, genomic resource, phylogenetic relationship, East Asia

## Abstract

To address limited genomic resources hindering *Hypopterygium* phylogenetics/taxonomy, the chloroplast genome of *Hypopterygium flavolimbatum* Müll. Hal. (Hypopterygiaceae, Bryophyta) was sequenced. The chloroplast genome is 125,283 bp in length, comprising an 86,845 bp large single-copy region, an 18,606 bp small single-copy region, and a pair of 9916 bp inverted repeats. A total of 127 genes were predicted, including 82 protein-coding genes, 36 tRNA genes, and 8 rRNA genes. Phylogenetic analysis indicates that *H. flavolimbatum* exhibits the closest genetic affinity with a clade containing *Myurella julacea*, *Myuroclada maximowiczii*, and *Climacium dendroides*. This study provides essential sequence data for species identification and phylogenetics.

## Introduction

*Hypopterygium flavolimbatum* Müll. Hal. is a bryophyte and member of the Hypopterygiaceae family that is widely distributed across East Asia, including China, Japan, and Nepal (Kruijer 2002; Zuo et al. 2015). This species plays a vital ecological role in forest ecosystems by providing dense ground cover that stabilizes soil moisture and prevents erosion. While previous studies have thoroughly characterized its morphology (Wu et al. 2024) and molecular traits (Zuo et al. 2015), the absence of a complete chloroplast genome has hindered deeper genomic insights into the *Hypopterygium* genus.

In this study, we sequenced the complete chloroplast genome of *H. flavolimbatum* to establish a genomic benchmark for this genus. This dataset advances comparative studies on bryophyte evolution and underscores the ecological role of this species in sustaining forest ecosystems in East Asia.

## Materials and methods

**Figure 1.**
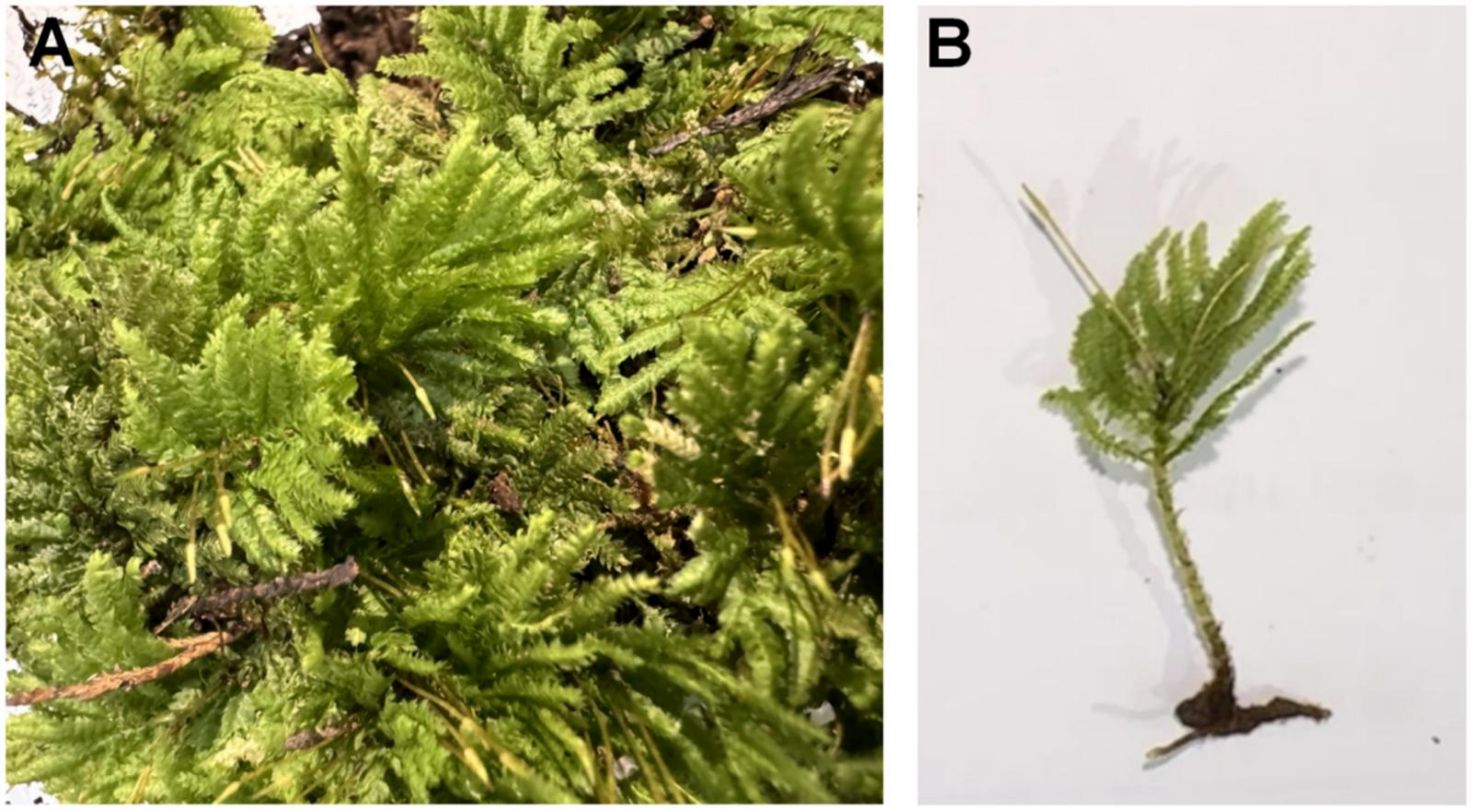
*Hypopterygium flavolimbatum*. (A) Image showing the whole plant, including creeping growth habit and pinnate branching. (B) Shoot apex with spirally arranged ovate leaves and finely serrated margins. Both photographs were taken by the author of this article, Zheng-Kai Ye.

In October 2024, specimens of *H. flavolimbatum* were collected from Chengdu, China (30.69° N, 104.05° E), and was identified by Dr. Xiang-Hua Fang. A single specimen was deposited in the Jingning County Ecological Forestry Development Center (voucher number S2024-10-03; contact: Zheng-Kai Ye, 1076912852@qq.com; Figure 1). Genomic DNA was extracted from leaf tissue samples using a Rapid Plant Genomic DNA Isolation Kit (Sangon, Shanghai, China). Sequencing was performed utilizing the Illumina HiSeq 2500 platform, generating 150-bp paired-end reads. The genomic sequences were subsequently screened and assembled with the GetOrganelle software (Jin, Yu, et al. 2020). The coverage depth of the genome was determined using SAMtools v1.16.1 (Li et al. 2009), and the sequencing depth and coverage map was drawn using ggplot2 (Ito and Murphy 2013) in R (Figure S1). Annotation of the chloroplast genome was carried out using the online platforms GeSeq (Tillich et al. 2017) and CPGAVAS2 (Shi et al. 2019), which facilitated the identification of the starting position of the chloroplast genome, the IR regions, and the annotation of genes. We further used CPGView to improve the annotation, visualize the structure of the chloroplast genome, and identify gene structures, including cis-splicing and trans-splicing (Liu et al. 2023).

A phylogenetic analysis was conducted using the chloroplast genomes of 20 Bryopsida species, with *Sanionia uncinata* designated as the outgroup taxon. We selected *S. uncinata* as the outgroup following its common use in bryophyte chloroplast phylogenomics and its phylogenetic position outside our ingroup (*Hypopterygium* spp.). In particular, Park et al. (2018) sequenced the complete chloroplast genome of *S. uncinata* and used it in comparative phylogenetic analyses of moss plastomes, demonstrating its suitability as a well-characterized external reference taxon. The chloroplast genome sequences were aligned by using MUSCLE version 3.8.31, utilizing its default settings (Edgar 2004). The maximum likelihood method was implemented for the phylogenetic analysis using RAxML (Stamatakis 2006), incorporating 1000 bootstrap replicates and the GTR + G + I substitution model. The R package ggtree (Xu et al. 2022) was used to visualize the phylogenetic tree.

## Results

**Figure 2.**
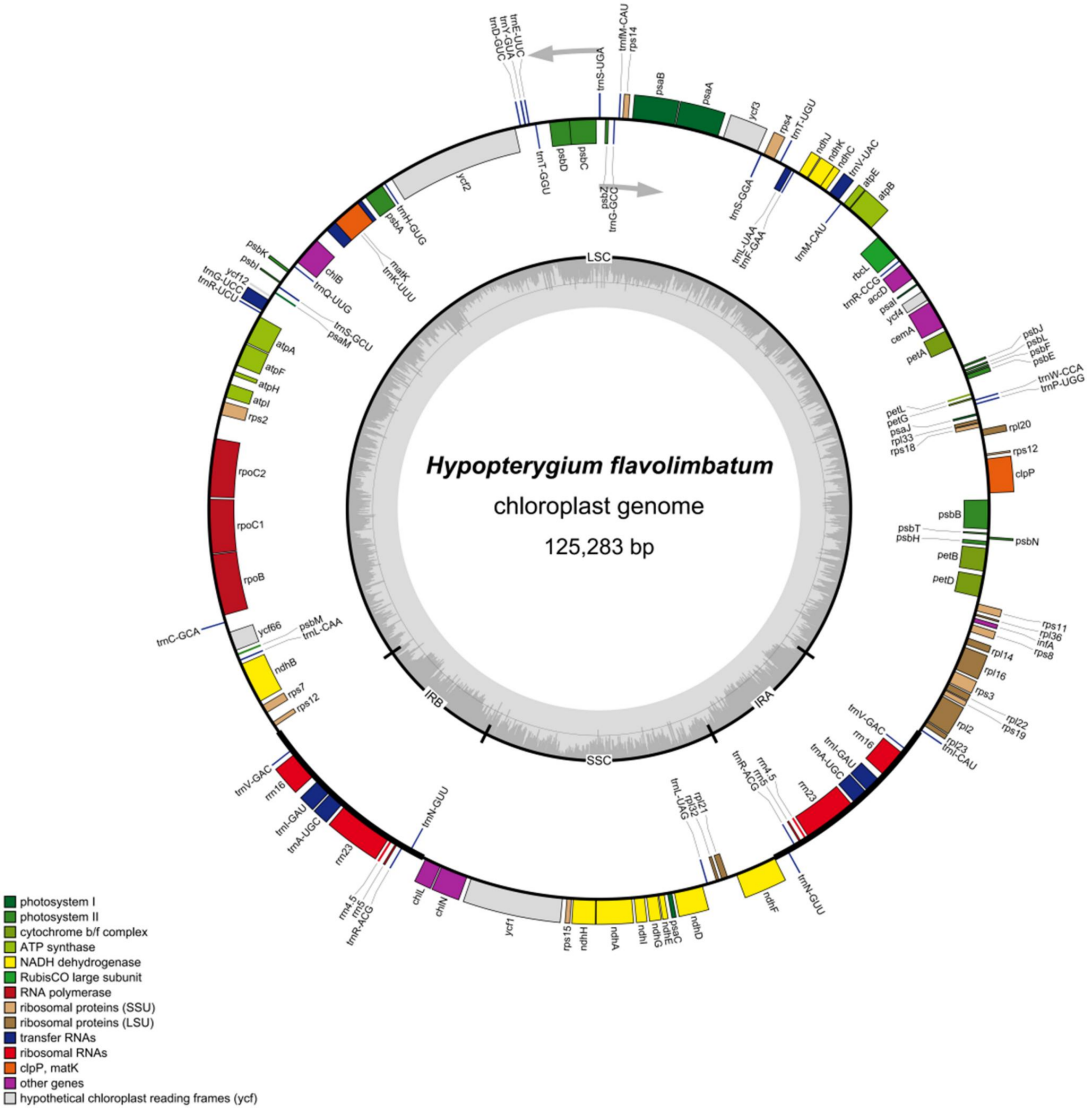
*Hypopterygium flavolimbatum* chloroplast genome map. The inner circle shows the GC (dark grey) and AT (light grey) contents. Different colors on the outer circle indicate different functional gene classifications.

The complete chloroplast genome sequence of *H. flavolimbatum* is 125,283 bp long. It comprises a large single-copy (LSC) region of 86,845 bp, two short-inverted repeat regions (IRA and IRB) of 9916 bp each, and a small single-copy (SSC) region of 18,606 bp. The overall GC content of the chloroplast genome was 28.82%. Within the chloroplast genome, 127 distinct genes were identified, including 82 protein-coding genes, 8 ribosomal RNA genes, and 36 transfer RNA genes (Figure 2). Furthermore, 11 cis-spliced genes—namely *rpl2*, *rpl16*, *petD*, *petB*, *clpP*, *ycf3*, *atpF*, *rpoC1*, *ycf66*, *ndhB*, and *ndhA*—were confirmed and annotated using multiple sequence alignments (Figure S2). Notably, the *rps12* gene within the chloroplast genome of *H. flavolimbatum* exhibits a characteristic split structure, a trait frequently observed in plant chloroplast genomes (Figures 2 and S3).

**Figure 3.**
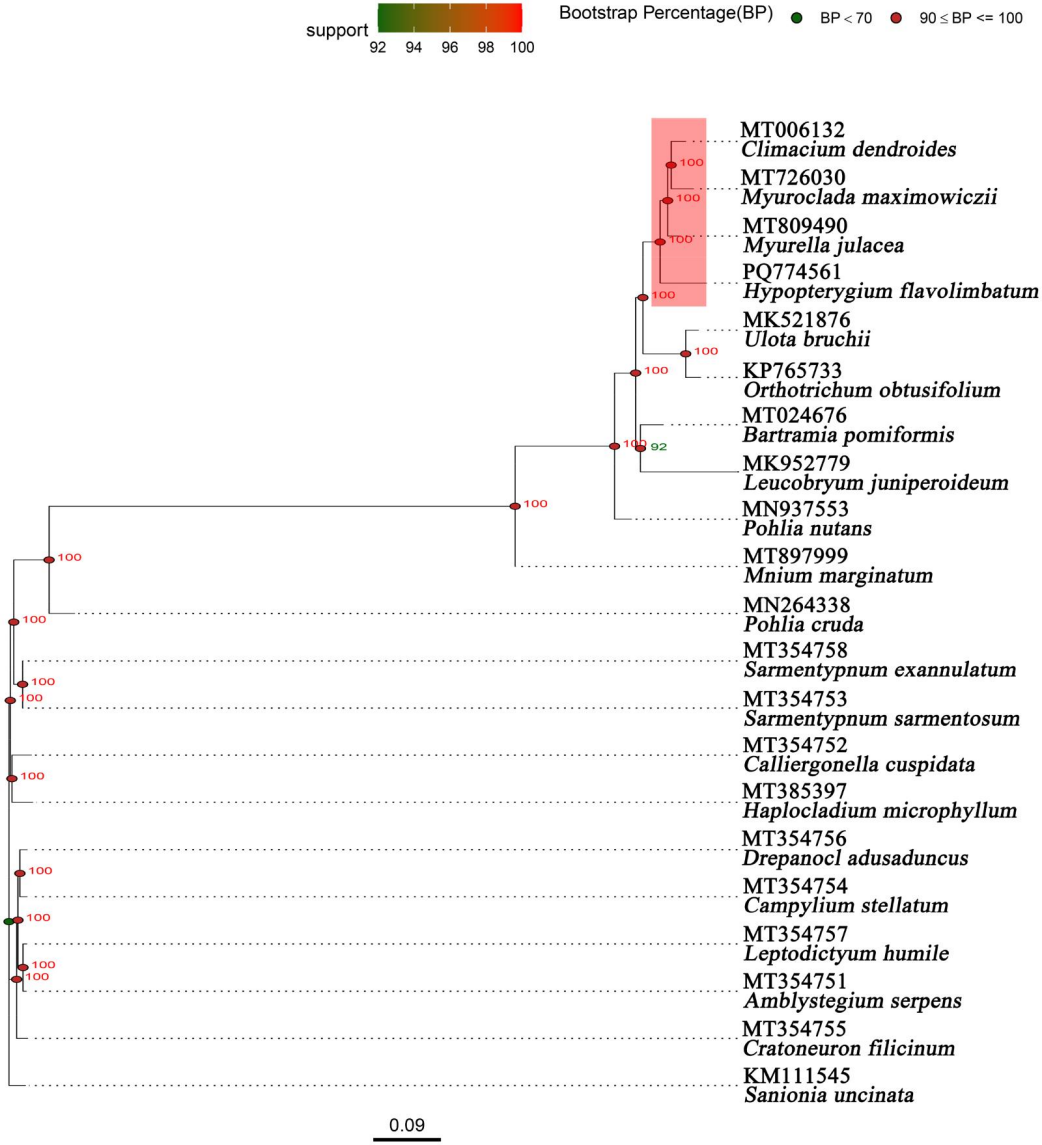
Phylogenetic tree showing chloroplast genome sequences of *Hypopterygium flavolimbatum* and 20 other species. Bootstrap values are indicated at the nodes, while species names and GenBank accession numbers are displayed at the termini of each branch. The horizontal scale denotes genetic distance, with one unit equivalent to 0.09. The following sequences were used: *Climacium dendroides* (MT006132) (Han, Choi, et al. 2020), *Myuroclada maximowiczii* (MT726030) (Han, Choi, et al. 2020), *Myurella julacea* (MT809490) (Han, Park, Yoon, et al. 2020), *Hypopterygium flavolimbatum* (PQ774561), *Ulota bruchii* (MK521876) (unpublished), *Orthotrichum obtusifolium* (KP765733) (unpublished), *Bartramia pomiformis* (MT024676) (Han, Park, Choi, et al. 2020), *Leucobryum juniperoideum* (MK952779) (Min et al. 2019), *Pohlia nutans* (MN937553) (Jin, Zhang, et al. 2020), *Mnium marginatum* (MT897999) (unpublished), *Pohlia cruda* (MN264338) (Zhang et al. 2019), *Sarmentypnum exannulatum* (MT354758) (Sheng et al. 2020), *Sarmentypnum sarmentosum* (MT354753) (Sheng et al. 2020), *Calliergonella cuspidata* (MT354752) (Sheng et al. 2020), *Haplocladium microphyllum* (MT385397) (Mao et al. 2020), *Drepanocladus aduncus* (MT354756) (Sheng et al. 2020), *Campylium stellatum* (MT354754) (Sheng et al. 2020), *Leptodictyum humile* (MT354757) (Sheng et al. 2020), *Amblystegium serpens* (MT354751) (Sheng et al. 2020), *Cratoneuron filicinum* (MT354755) (Sheng et al. 2020), and *Sanionia uncinata* (KM111545) (Park et al. 2018).

A maximum-likelihood phylogenetic tree incorporating 20 Bryopsida species was developed for *H. flavolimbatum*. The results revealed that *H. flavolimbatum* is a sister of a clade containing *Myurella julacea*, *Myuroclada maximowiczii*, and *Climacium dendroides* (bootstrap = 100; Figure 3), suggesting shared evolutionary innovations within this lineage. This clarifies the placement of *H. flavolimbatum* within the Hypnobryales order (Buck et al. 2008).

## Discussion and conclusion

This study presents the complete chloroplast genome of *H. flavolimbatum*, which represents the first genomic resource of the genus *Hypopterygium*, addressing a critical gap in bryophyte molecular research. The plastome structure aligns with typical bryophyte chloroplast genomes, characterized by a quadripartite organization and moderate GC content (28.82%), comparable with other mosses such as *M. julacea* (Han, Park, Yoon, et al. 2020) and *C. dendroides* (Han, Choi, et al. 2020). The presence of 127 genes, including 11 cis-spliced genes and the fragmented *rps12* gene, underscore the conserved structural features shared across Bryopsida (Mao et al. 2020; Han, Choi, et al. 2020). These findings corroborate previous observations that split genes and trans-splicing mechanisms are widespread in plant plastomes, likely facilitating functional plasticity in non-vascular plants.

Phylogenetic analysis revealed that *H. flavolimbatum* is a sister to a clade containing *M. julacea*, *M. maximowiczii*, and *C. dendroides* (bootstrap = 100), consolidating its placement within the Hypnobryales order. This close affinity suggests shared evolutionary adaptations potentially linked to ecological niches in East Asian forest ecosystems. These results are consistent with morphological studies highlighting similarities in leaf architecture and growth habits among Hypnobryales species (Kruijer 2002; Zuo et al. 2015).

The ecological significance of *H. flavolimbatum* as a soil stabilizer and moisture regulator underscores the urgent need for genomic conservation efforts. By providing a reference genome, this study will facilitate future investigations into adaptive traits, such as stress tolerance genes, and the mechanisms underlying ecological resilience. Furthermore, the dataset enables comparative analyses across bryophyte lineages, offering insights into chloroplast genome evolution in non-vascular plants.

In conclusion, this study advances the genomic understanding of *Hypopterygium* and provides a foundational resource for bryophyte phylogenetics. By bridging molecular and ecological research, this study emphasizes the importance of conserving understudied bryophytes that play pivotal roles in maintaining the integrity of forest ecosystems.

## Supplementary Material

Supplemental Material

## Data Availability

The genome sequence data supporting this study are openly available in GenBank of NCBI at https://www.ncbi.nlm.nih.gov under the accession number PQ774561. The associated BioProject, SRA, and Biosample numbers are PRJNA1199712, SRR31755810, and SAMN45886260, respectively.
