# Supplementary material for: Complete chloroplast genome analysis of Hypopterygium flavolimbatum Müll. Hal. (Hypopterygiaceae, Bryophyta)
Source: Mitochondrial DNA B Resour. 2025 Jul 22;10(8):758–62. doi: 10.1080/23802359.2025.2535629 (PMC12284983; doi:10.1080/23802359.2025.2535629)
Supplement: Supplemental Material [file TMDN_A_2535629_SM6137.docx]

**Supplemental Material**


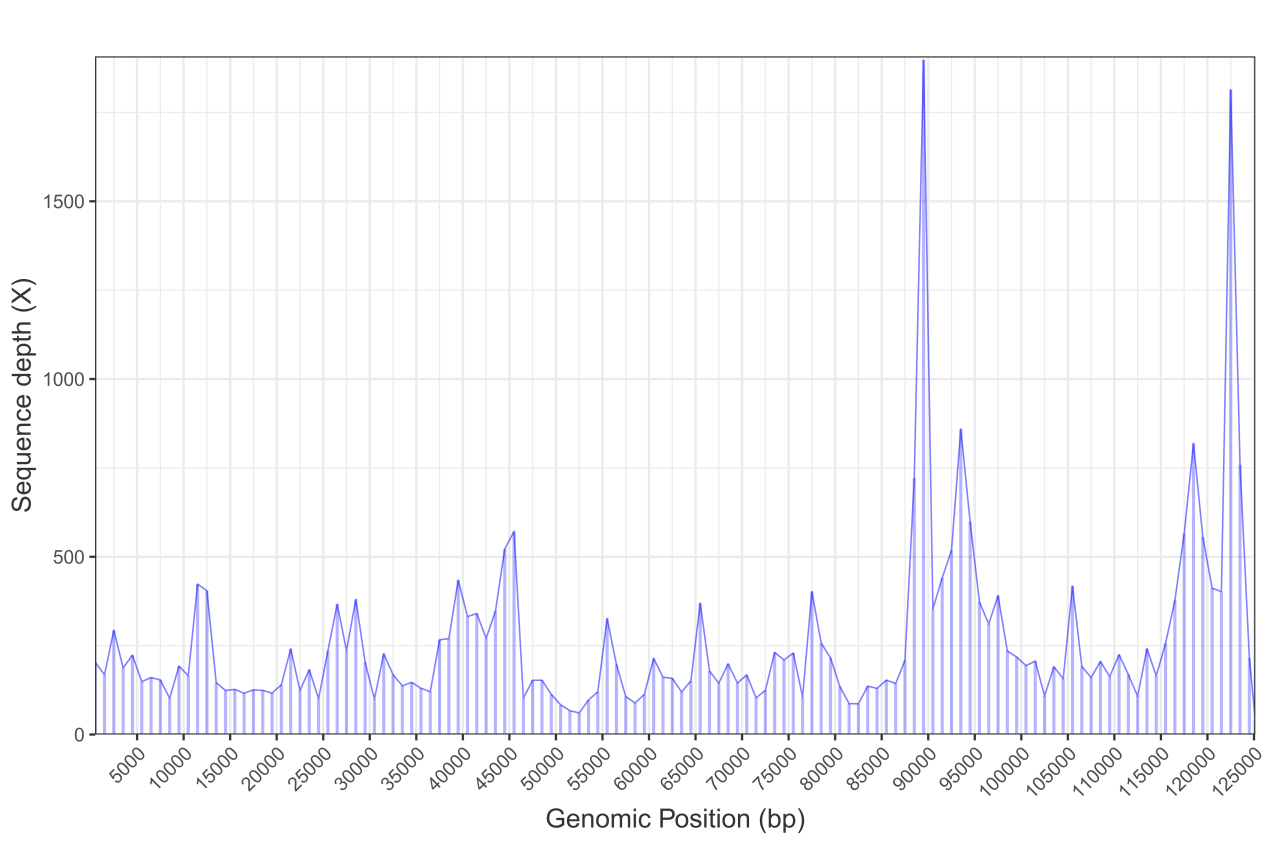


**Figure S1.** The distribution of chloroplast genome sequencing depth for *Hypopterygium flavolimbatum* is depicted in the graph, with the horizontal axis representing genomic position and the vertical axis indicating sequencing depth.

**
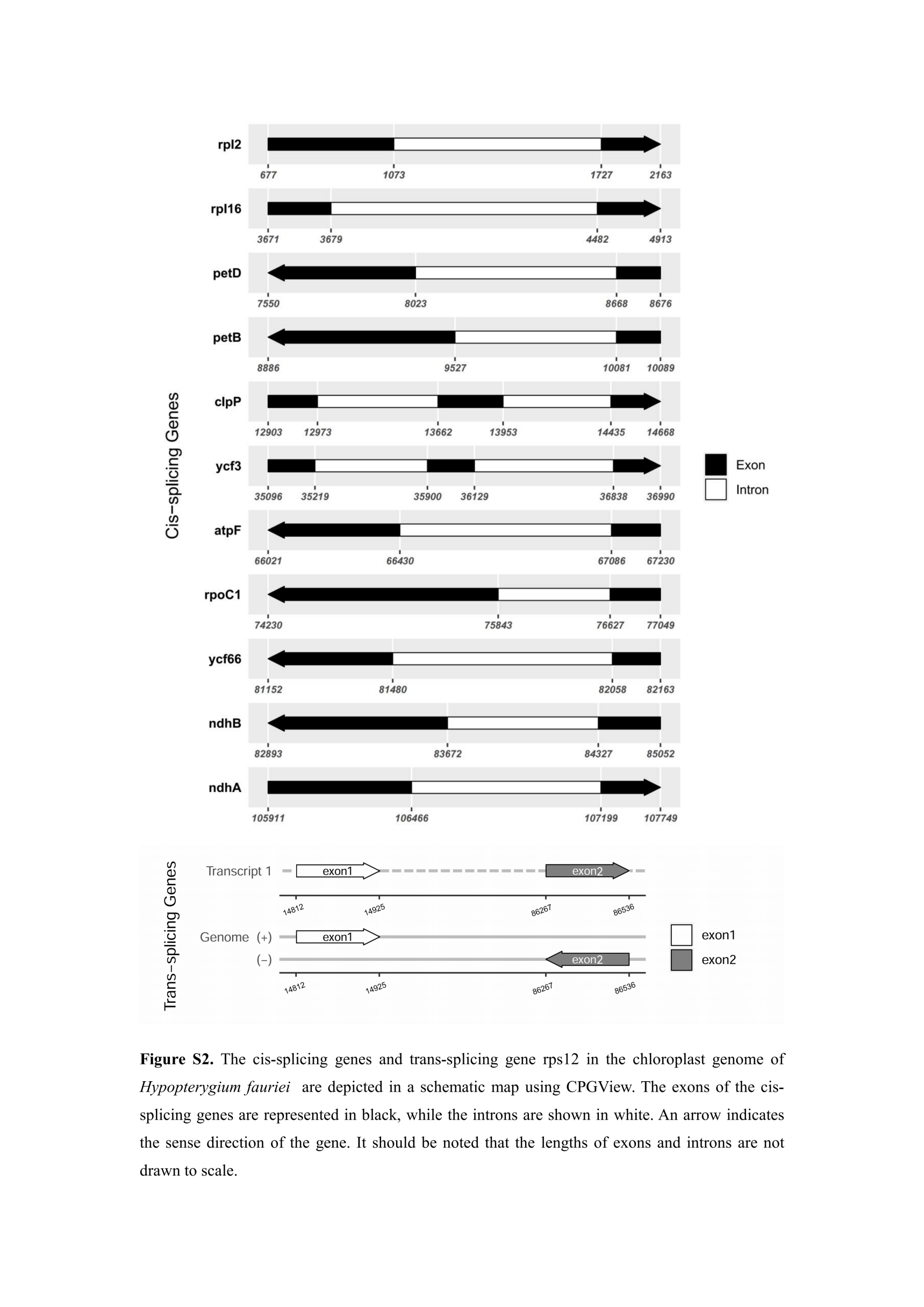
**

**Figure S2.** The cis-splicing genes in the chloroplast genome of *Hypopterygium flavolimbatum* are depicted in a schematic map using CPGView. The exons of the cis-splicing genes are represented in black, while the introns are shown in white. An arrow indicates the sense direction of the gene. It should be noted that the lengths of exons and introns are not drawn to scale.

**
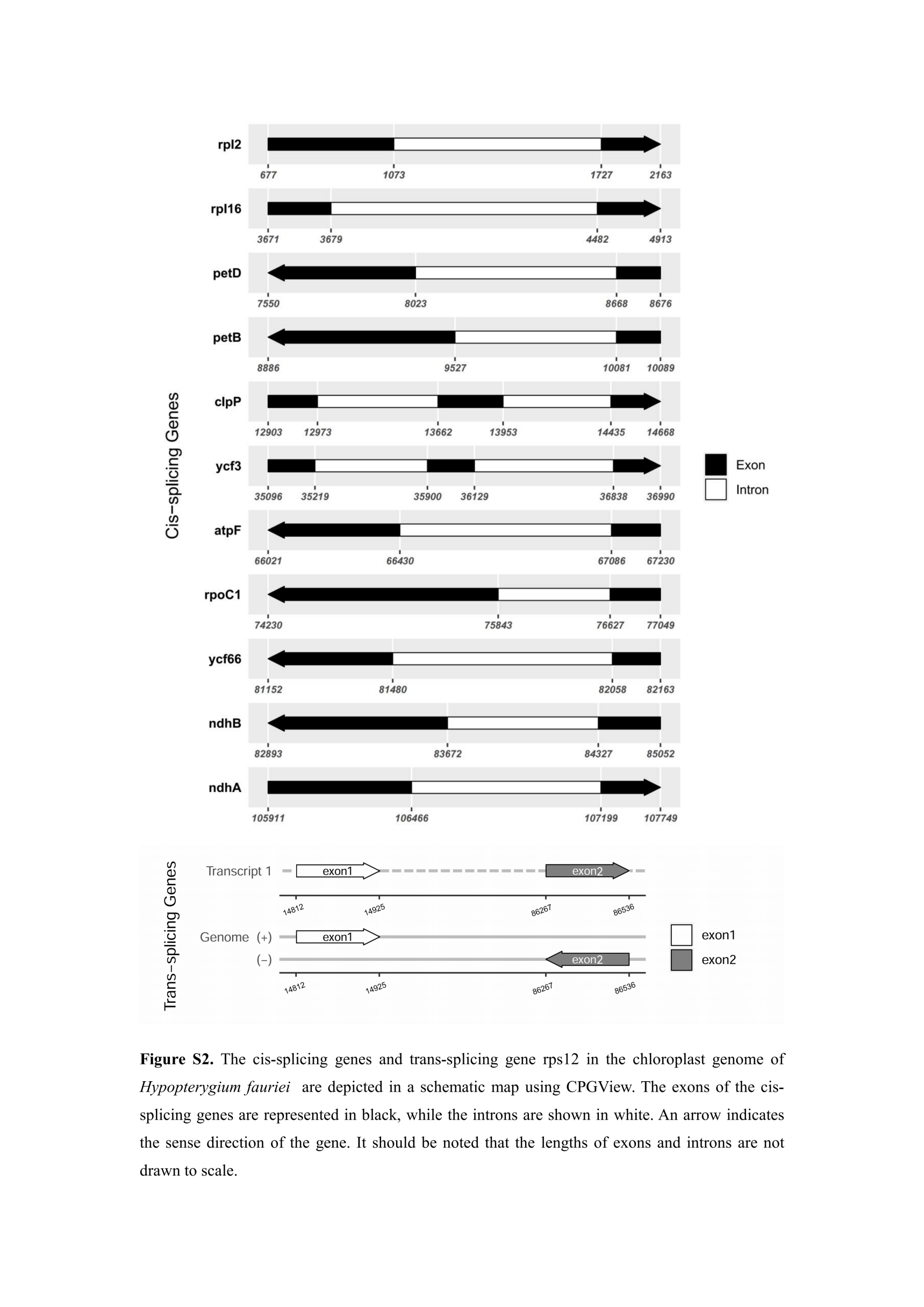
**

**Figure S3.** The trans-splicing gene *rps12* in the chloroplast genome of *Hypopterygium flavolimbatum* are depicted in a schematic map using CPGView. An arrow indicates the sense direction of the gene. It should be noted that the lengths of exons are not drawn to scale.
